# Fenton-Mediated Biodegradation of Chlorendic Acid – A Highly Chlorinated Organic Pollutant – By Fungi Isolated From a Polluted Site

**DOI:** 10.3389/fmicb.2019.01892

**Published:** 2019-08-14

**Authors:** Inge Jambon, Sofie Thijs, Giselle Torres-Farradá, François Rineau, Nele Weyens, Robert Carleer, Pieter Samyn, Jaco Vangronsveld

**Affiliations:** ^1^Centre for Environmental Sciences, Hasselt University, Diepenbeek, Belgium; ^2^Department of Microbiology and Virology, Faculty of Biology, University of Havana, La Habana, Cuba; ^3^Institute for Materials Research, Hasselt University, Diepenbeek, Belgium; ^4^Department of Plant Physiology, Faculty of Biology and Biotechnology, Maria Curie-Skłodowska University, Lublin, Poland

**Keywords:** bioremediation, chlorendic acid, Fenton reaction, fungi, plant growth promoting bacteria, radicals

## Abstract

Chlorendic acid is a recalcitrant, highly chlorinated organic pollutant for which no microbial degrader has yet been identified. To address this knowledge gap, fungi were isolated from bulk soil, rhizosphere, and roots of the common bent (*Agrostis capillaris*) and the hybrid poplar [*Populus deltoides* × (*Populus trichocarpa* × *P. deltoides*) cv. Grimminge], both of which grow on a chlorendic acid polluted site in Belgium. Isolates were taxonomically identified and phenotypically screened for chlorendic acid degradation. Several fungal isolates could degrade chlorendic acid in liquid media up to 45%. The chlorendic acid degrading fungal isolates produced higher levels of hydroxyl radicals when exposed to the pollutant when compared to non-exposed controls, suggesting that the oxidative degradation of chlorendic acid occurs through production of Fenton-mediated hydroxyl radicals. In addition, the isolated Ascomycete *Penicillium* sp. 1D-2a degraded 58% of the original chlorendic acid concentration in the soil after 28 days. This study demonstrates that the presence of fungi in a chlorendic acid polluted soil can degrade this highly chlorinated organic pollutant. These results indicate that recalcitrant, seemingly non-biologically degradable organic pollutants, such as chlorendic acid, can be remediated by using bioremediation, which opens new perspectives for *in situ* bioremediation.

## Introduction

Chlorendic acid, also known as HET acid (hexachloro-endo-methylenetetrahydronaphthalic or 1,4,5,6,7,7-hexachlorobicyclo[2.2.1]-hept-5-ene-2,3-dicarboxylic acid), is a highly chlorinated dicyclic organic acid. It has a molecular weight of 388.8 g/mol, melting point of 208-201°C, log Kow of 2.3, water solubility of 3.5 g/l and vapor pressure of 1.4 × 10^–8^ mm Hg at 25°C. Chlorendic acid is synthesized using a Diels-Alder reaction in a closed system (IPCS, 1996). There are several industrial applications of the compound, e.g., chlorendic acid is used as a flame retardant in plasticizers, coatings, resins, polyurethane foams and wool fabrics. It is also used in the manufacturing of alkyl resins, polyester resins for paints, inks, plastics, and in the manufacturing of corrosion resistant piping, scrubbers and tanks, and as an extreme pressure lubricant (HSDB, 2017). Even though chlorendic acid is produced in a closed system, it can be released in the environment via hydrolytic degradation of the polyesters after their disposal. In addition, flame-proofing processes in the textile industry can lead to chlorendic acid contaminated wastewater (IPCS, 1996). Further, the oxidation of chlorinated cyclopentadiene pesticides in the environment such as endosulfan, chlordane, heptachlor, dieldrin, and aldrin (IPCS, 1996) can give rise to chlorendic acid polluting the soil and (ground) water. Health studies showed that chlorendic acid is irritating for the skin, eyes and respiratory tract (HSDB, 2017). It is also anticipated to be a human carcinogen based on studies in experimental animals (NTP, 2011). Oral exposure to chlorendic acid caused tumors in rats and mice in several tissues including liver, pancreas, and lungs (NTP, 1987).

In the United States, chlorendic acid was detected in landfill leachate at concentrations up to 455 mg l^–1^ (IPCS, 1996). It is quite persistent in the environment because of its high chlorination and the fact that it has poor electron donor properties (Sebastian et al., 1996; Ndokiari, 2013). In addition, chlorendic acid has low soil binding potential and therefore expected to have a high mobility. Despite this, it is not expected to volatilize from soil or water (NTP, 2011). The only degradation route known of chlorendic acid is by chemical oxidation such as ozonation, UV-radiation or gamma irradiation, leading ultimately to the release of HCl, formic acid, chlorate, and the formation of chlorendic acid anhydride (Freshour et al., 1996; Sebastian et al., 1996; Ndokiari, 2013; Hermes and Knupp, 2015; Shah et al., 2015). However, we are interested to know if bioremediation, and not physical or chemical treatments, is able to treat chlorendic acid polluted soils.

Bioremediation is a cost-effective, environmentally friendly alternative to physicochemical techniques for cleaning up polluted environments. Soil bacteria and fungi can be harnessed for their potential to improve the bioremediation of organic pollutants (Jambon et al., 2018). Bacteria can co-metabolically degrade pollutants or use it as a sole carbon and energy source during catabolism or mineralization (Barac et al., 2004; Wenzel, 2009; Weyens et al., 2009; Glick, 2010). Also fungi possess capacities to degrade organic pollutants. In fact, soil fungi are often more able to degrade highly chlorinated xenobiotics than bacteria. In contrast to bacteria, fungi form extended mycelial networks to penetrate soil pores, organic matter, and rock matrices. These mycelial networks have the additional benefit of bringing the degrader into closer contact with the soil pollutants (Harms et al., 2011). A remarkable characteristic of white-rot, saprotrophic and mycorrhizal fungi is their capacity to exude a broad spectrum of oxidative enzymes that can co-metabolize organic pollutants (Hofrichter and Ullrich, 2010), often much more powerful and more broad substrate-range enzymes compared to bacteria. These enzymes, such as phenoloxidases, laccases and peroxidases show a low substrate specificity, which makes them of high interest for application in bioremediation (Singh Arora and Kumar Sharma, 2010; Viswanath et al., 2014; Torres-Farradá et al., 2017, 2018). Fungi can also generate hydroxyl radicals via secretion of iron reducing compounds and following the induction of Fenton chemistry (Hammel et al., 2002). This mechanism is mostly studied in brown-rot fungi in which the hydroxyl radicals oxidize lignified plant cell walls (Koenigs, 1974; Hyde and Wood, 1997; Arantes et al., 2011, 2012), but is also described for white-rot fungi and ectomycorrhizal fungi (Arantes et al., 2011; Rineau et al., 2012; Shah et al., 2015). Hydroxyl radicals are strong, non-selective oxidative agents that can “attack” the pollutant by the abstraction of a functional groups (hydrogen and chloride) or the addition of a hydroxyl radical to the organic pollutant after which it can more easily degrade into more easily metabolizable by-products (Freshour et al., 1996). Fungal degradation of organic pollutants via the production of Fenton-mediated hydroxyl radicals has been described several times previously (Kerem et al., 1999; Schlosser et al., 2000; Newcombe et al., 2002; Kramer et al., 2004; Purnomo et al., 2008; Krueger et al., 2015).

The potential of chlorendic acid biodegradation appeared to be very limited based on the available literature. For example, the removal of chlorendic acid was previously investigated in an industrial landfill leachate (Ying et al., 1986), and in a batch activated sludge reactor (Ying et al., 1987), but both studies could not attribute removal of chlorendic acid to biodegradation. Because any information is lacking on the biodegradability of chlorendic acid, the goal of this study was to evaluate the possibility of chlorendic acid degradation by fungal, and bacterial isolates in bioremediation. To this end, bacteria and fungi were isolated from an industrial site with historical chlorendic acid contamination and screened for their degradation potential. Furthermore, the pathways involved in the degradation were investigated including discriminating passive sorption from degradation, comparing enzymatic and non-enzymatic degradation mechanisms, and evaluating phytotoxicity of the resulting medium after fungal transformation. Since chlorendic acid is structurally related to a number of chlorinated insecticides such as dieldrin, endrin, chlordane, endosulfan and heptachlor (NTP, 1987), the outcomes of this research has also implications for application in bioremediation of other more frequently occurring pollutants.

## Materials and Methods

### Study Site and Sampling

**FIGURE 1:**
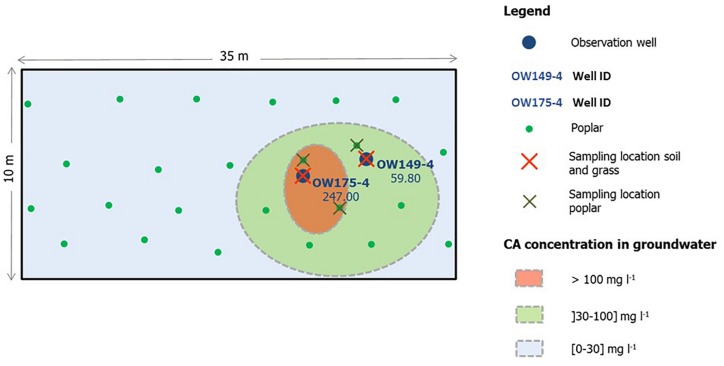
Schematic overview of the sampling site and sampling locations. Soil samples (surface soil, –1, –1.5, and –2 m) and samples of root and rhizosphere of grass were taken in an area of one meter around OW149-4 and OW175-4 (*n* = 4). Root and rhizosphere samples of poplar were taken of the trees growing closest to the two observation wells, with four repetitions for each tree.

The study site is an industrial site located in Genk, Belgium (50°55′55.8′′N; 5°28′29.6′′E) historically polluted with chlorendic acid, monochlorobenzene and phenol, as a result of industrial activities and improper waste release. Soil characteristics indicate a moderate dry sandy soil with 1% organic matter and average dry matter content of 86.3%. The natural vegetation consists mainly of grasses (dominated by *Agrostis capillaris*), black cherry (*Prunus serotina*) and Scotch broom (*Cytisus scoparius*). Genk has an annual rainfall of 852 mm per square meter, an average annual temperature of 2.8°C in January and 18.5°C in July (Royal Meteorological Institute Belgium). Several observation wells are distributed over the site and are used to monitor the concentrations of the pollutants in the groundwater. Based on these measurements, a gradient in chlorendic acid pollution was observed (Figure 1). In March 2013, 24 4-m long hybrid poplar cuttings of the Grimminge cultivar [*Populus deltoides* × (*Populus trichocarpa* × *Populus deltoides*)] were planted on the field for future phytoremediation purposes. The trees were distributed over a defined rectangular grid of 10 m by 35 m, enclosing the area with the highest concentrations of chlorendic acid in the groundwater (>100 mg l^–1^) (Figure 1). In 2015, samples for isolation of cultivable fungal strains were collected from soil, rhizosphere and roots of common bent and hybrid poplar. Four soil samples were taken in a 1-m perimeter around observation wells OW175-4 and OW149-4 (Figure 1), from surface soil, −1, −1.5, and −2 m. Common bent rhizosphere and roots were sampled inside the 1-m perimeter around each of the observation wells. Poplar rhizosphere and root samples were taken from three trees growing in the zones with high concentrations of chlorendic acid (Figure 1). From each tree, four sample repetitions were taken. In total, 32 soil samples were collected (4 × 4 depths × 2 locations), 8 bentgrass root and rhizosphere samples (4 × 2 locations) and 12 poplar rhizosphere and root samples (4 × 3 trees). Soil samples were taken with sterile tools and transported in sterile bags. Roots were collected with soil particles still adhering to the surface, which was considered the rhizosphere fraction. For poplar, the tertiary fine roots were selected. Roots with adhering rhizosphere fraction were obtained with sterile tools and directly transferred into falcon tubes with 30 ml sterile 10 mM MgSO_4_.

### Isolation of Bacteria and Fungi

Soil samples were homogenized and vortexed in sterile 10 mM MgSO_4_ prior to isolations. Root and rhizosphere samples were vortexed after which the roots were removed from the MgSO_4_. The resulting soil pellet after centrifugation (15 min, 2700*g*) constituted the rhizosphere fraction. Poplar roots were surface sterilized by immersing them for 5 min in 1% NaClO and bunchgrass roots were sterilized with 0.1% NaClO supplemented with 0.1% Tween. Following treatment, all roots were rinsed four times in sterile distilled water. The last rinsing solution was plated on 869 medium (Mergeay et al., 1985) to confirm surface sterility by scoring microbial growth after 7 days of incubation at 30°C. The surface sterilized roots were assembled in 15 ml sterile 10 mM MgSO_4_ and mixed for 1 min using a Polytron PR1200 mixer (Kinematica A6).

For fungi isolations, dilutions up to 10^–4^ of the soil, rhizosphere and endophytic root solutions were plated onto peptone-glucose-acid-agar medium pH 5, as described by Dietrich and Lamar (1990), and incubated for 7 days at 23°C. After incubation, all fungal mycelia were selected, purified and stored on solid malt extract [2% (w/v)]-peptone [0.3% (w/v)] medium pH 5 at 4°C.

For bacteria isolation, dilutions of the soil samples were added to a selective minimal medium (Sebastian et al., 1996) supplemented with 25 mg l^–1^ chlorendic acid as sole carbon source, or with 3 mM glucose in addition to chlorendic acid. Every 10 days, 10 ml of the culture was transferred into 90 ml of fresh medium with a slightly higher concentration of chlorendic acid, ultimately leading to 60 mg l^–1^ chlorendic acid after 3 months of incubation at 30°C and 120 rpm. At the end of the experiment, chlorendic acid concentrations were determined using High Performance Liquid Chromatography (HPLC). The surviving bacterial strains were purified and stored in a glycerol solution [15% (w:v) glycerol; 0.85% (w:v) NaCl] at −40°C.

### Identification of Isolated Bacteria and Fungi

DNA was extracted from bacterial isolates using the DNeasy^®^ Blood and Tissue kit (Qiagen, Venlo, Netherlands) and from fungal isolates using the DNeasy^®^ PowerSoil^®^ kit (Qiagen, Venlo, Netherlands) following the manufacturer’s instructions with minor adaptations. The 10 min vortex step was replaced with a 20 min shredding step at 30 Hz using a Retsch MM400 grinding mill (Retsch, MA, United States). The DNA yield and purity of the extracts were evaluated using a NanoDrop spectrophotometer (NanoDrop Technologies Inc., Wilmington, DE, United States).

For bacterial samples, the 16S rRNA gene were amplified using the bacteria-specific 27F primer (5′-AGAGTTTGAT CCTGGCTCAG-3′; Schauer and Hahn, 2005) and the universal prokaryotic 1392R primer (5′-ACGGGCGGTGTGTRC-3′; Amann et al., 1995) under following cycling conditions: an initial denaturation at 95°C for 5 min, followed by 35 cycles of denaturation at 94°C for 1 min, annealing at 52°C for 30 s and extension at 72°C during 3 min, ending with a final extension phase at 72°C for 10 min. Reactions were carried out in 50 μl reaction volumes containing 1× High Fidelity PCR buffer, 2 mM MgSO_4_, 0.2 mM of each dNTP, 0.2 μM of each primer, 1.25 U High Fidelity Taq DNA-polymerase, and 1 μl of template DNA.

For fungal identification, the ITS-2 rRNA gene was amplified, using the primer pair ITS86F binding to the 5.8S of the rRNA operon (5′-GTGAATCATCGAATCTTTGAA-3′) and ITS4R (5′-TCCTCCGCTTATTGATATGC-3′) under the following cycling conditions: an initial denaturation at 95°C for 2 min, followed by 40 cycles of denaturation at 95°C for 30 s, annealing at 55°C for 30 s and extension at 72°C for 1 min, ending with a final extension at 72°C for 10 min (Op De Beeck et al., 2014). Reactions were carried out in 50 μl reaction volumes containing 1× High Fidelity PCR buffer with 1.8 mM MgCl_2_, 0.2 mM of each dNTP, 0.4 μM of each primer, 1.25 U High Fidelity Taq DNA-polymerase, and 1 μl of template DNA. The PCR products were checked for their size and purity on a 1.5% (w/v) agarose gel by comparing the amplicon bands with a 1 kb ladder.

Amplicons were bidirectionally Sanger sequenced at Macrogen (Amsterdam, Netherlands). Reads were quality filtered using Geneious v.4.8.5 and consensus sequences were generated (Kearse et al., 2012). Identification was performed by comparing the acquired sequences with reference sequences in the Ribosomal Database Project (RDP, release 11.4, Wang et al., 2007) for bacterial sequences and in the UNITE database v7.0 (Koljalg et al., 2014) or MycoBank database (Robert et al., 2013) for fungal reads. Sequences were aligned using ClustalW in MEGA X (Kumar et al., 2018), and an evolutionary tree was inferred by using the Maximum Likelihood method and Tamura-Nei model (Tamura and Nei, 1993), with 500 bootstrap replicates.

### Screening for Plant Growth-Promoting Bacteria

The isolated bacteria were screened for their potential to promote plant growth *in vitro*. For this, the bacterial strains were cultivated in liquid 869 medium for 3 days at 30°C, washed twice in 10 mM MgSO_4_ and subsequently used for inoculation in the specific media for plant growth-promotion tests. In brief, bacterial phosphate solubilization was tested using plates containing NBRIP growth medium (Nautiyal, 1999) and with holes of 0.5 cm diameter, in which 10 μl of bacterial suspension was inoculated. After 1 week at 30°C, a halo formation around the holes indicated phosphate solubilization. To test for nitrogenase activity, bacteria were inoculated in a nitrogen-free semi-solid malate-sucrose medium containing bromothymol blue (3 ml l^–1^) as a pH-indicator (Xie et al., 2006). The same medium containing 0.12 g l^–1^ NH_4_Cl was used as a positive control. After 1 week incubation at 30°C, a color change from blue to yellow-green indicated bacterial anaerobic nitrogen fixation. Indole-3-acetic acid (IAA) production was evaluated by inoculating the bacterial cultures in 1 ml 1/10 strength 869 medium with 0.5 g l^–1^ L-tryptophan and incubating at 30°C. After 4 days, 1 ml of Salkowsky reagent was added to 0.5 ml of bacterial culture and a color change from yellow to pink indicated strains that produced IAA (Patten and Glick, 2002). Siderophores production was detected using the Chrome azurol S (CAS) reagent as described by Schwyn and Neilands (1987). For determining bacterial acetoin production, bacteria were inoculated in 1 ml of methyl red/Voges-Proskauer (MR/VP) and incubated for 48 h at 30°C. Subsequently, cultures were centrifuged and to 100 μl of supernatant, 10 μl of 50 mM of L-arginine, 10 μl alpha-naphtol (Barritz reagens, Sigma-Aldrich) and 25 μl of KOH (Barritz reagens, Sigma-Aldrich) were added (Romick and Fleming, 1998). After mixing, a color change to red indicated bacterial acetoin production. Production of organic acids was determined using the method described by Cunningham and Kuiack (1992). Activity of 1-aminocyclopropane-1-carboxylate (ACC) deaminase was detected by monitoring the amount of ketobutyrate produced by hydrolysis of ACC as described by Belimov et al. (2005). A color change from yellow to brown indicated strains positive for ACC deaminase activity.

### Screening for Chlorendic Acid-Degrading Fungi

The isolated fungi were screened for their capacity to degrade chlorendic acid in a minimal medium with chlorendic acid and glucose. First, the fungi were grown for 1 week at 23°C in liquid malt extract-peptone medium. Subsequently, the mycelia were washed twice in a minimal Ingestad medium pH 5 [Ingestad (1970, 1971, 1979)], containing 96 μM KNO_3_, 70 μM K_2_SO_4_, 63 μM KH_2_PO_4_, 58 μM K_2_HPO_4_, 732 μM NH_4_NO_3_, 36 μM Ca(NO_3_)_2_.4H_2_O, 62 μM Mg(NO_3_)_2_.6H_2_O, 13 μM HNO_3_, 5 μM H_3_BO_3_, 2 μM Mn(NO_3_)_2_.4H_2_O, 0.1 μM Zn(NO_3_)_2_.4H_2_O, 0.1 μM CuCl_2_.2H_2_O, 0.02 μM Na_2_MoO_4_.2H_2_O, and 3 μM Fe(NO_3_)_3_9H_2_O. Finally, the washed mycelia were incubated at 23°C for 2 weeks on Ingestad medium containing 50 mg l^–1^ chlorendic acid and 3 mM glucose. After 2 weeks, the concentrations of chlorendic acid in the media were determined using HPLC. All samples were tested in triplicate, including abiotic negative controls.

Samples for HPLC analyses were prepared by filtration of the supernatant through 0.22 μm PTFE filters (ref: 514-0068, VWR, Leuven, Belgium). Analyses were performed on an Agilent Technologies 1260 Infinity HPLC with a detection at 215 nm. The separation column used was an Acclaim 120 C18 Column, 2.1 mm × 100 mm, with 2.2 μm particle size and 120 Å pore size (Thermo Fisher Scientific, Waltham, MA, United States). HPLC grade water and acetonitrile were used as a mobile phase in a combination ratio of 50:25 for the first 5 min, 25:50 for the next 5 min and 0:75 for the rest of the run, with a flow rate of 0.4 ml min^–1^. Column temperature was kept at 40°C.

### Biosorption Experiments

To be able to distinguish degradation of chlorendic acid from adsorption onto the cell surfaces of the mycelia, the above-mentioned degradation experiment was repeated with an extra control of dead mycelia for each fungus. For this purpose, the mycelia were grown in liquid malt extract-peptone medium for 1 week at 23°C. For each isolate, three replicates were killed by immersing the mycelia overnight in 1% NaClO supplemented with 0.1% Tween, while three replicates were kept alive, after which all mycelia were washed twice with Ingestad medium. To confirm that fungal mycelia were dead, an FDA assay (fluorescein diacetate hydrolyzing activity, adapted from Lestan and Lamar, 1999) was performed on extra mycelia. Fluorescein diacetate reflects enzymatic activity and is hydrolyzed upon fungal activity to fluorescein. The fungal mycelia were transferred to 10 ml of 60 mM NaH_2_PO_4_ pH 7, to which 100 μl of 4.8 mM fluorescein diacetate solution in acetone was added. After 3 h of shaking at 37°C, a color change to fluorescent yellow was perceived if fungi were active. Dead fungal mycelia did not show any color change. After confirming the state of the fungal mycelia, they were immersed again in Ingestad medium containing 50 mgl^–1^ chlorendic acid and 3 mM glucose. After 2 weeks, the concentration of chlorendic acid in the media was determined for all samples by HPLC analyses, including an abiotic control condition (*n* = 3).

### Elucidation of Degradation Mechanism

#### Production of Ligninolytic Enzymes

The selected fungal isolates were examined for activity of ligninolytic enzymes laccase and peroxidase. They were cultivated in liquid malt-peptone medium for 1 week at 23°C and 120 rpm, subsequently washed twice in minimal Ingestad medium pH 5 and transferred to minimal Ingestad medium enriched with 50 mg l^–1^ chlorendic acid and 3 mM glucose and incubated for 2 weeks at 23°C and 120 rpm. The medium was sampled for determining laccase and peroxidase activities at the start of the experiment and every following day. Laccase activity was detected by monitoring the oxidation of 2,6-dimethoxyphenol at 468 nm using a Shimadzu UV-1800 spectrophotometer (Shimadzu, Kyoto, Japan), according to Bollag et al. (1988). Every reaction sample contained 930 μl of 100 mM acetate buffer at pH 5, 20 μl of 50 mM 2,6-dimethoxyphenol and 50 μl of sample. Non-specific peroxidase activity was measured by monitoring the oxidation of *o*-dianisidine at 460 nm, according to Claiborne and Fridovich (1979). Every reaction sample contained 500 μl of 100 mM acetate buffer at pH 5, 195 μl deionized water, 130 μl of 30.77 mM H_2_O_2_, 125 μl of 4 mM *o*-dianisidine, and 50 μl of sample. Enzymatic activity was expressed as the amount of enzyme needed to produce 1 μmol of product per minute at 23°C. A non-degrading fungal strain and abiotic controls were included. Every condition was tested in triplicate.

#### Production of Hydroxyl Radicals

The selected fungal isolates were also tested for their capacity to produce hydroxyl radicals. They were grown in liquid malt extract-peptone medium for 1 week at 23°C, subsequently washed twice in minimal Ingestad medium pH 5 and transferred to minimal Ingestad medium enriched with 50 mg l^–1^ chlorendic acid, 3 mM glucose and 2.5 mM terephthalate, and incubated for 2 weeks at 23°C. Samples were taken of the medium for measuring production of hydroxyl radicals at the start of the experiment and every following day. Production of hydroxyl radicals can be determined by using terephthalate as a probe, that produces fluorescent hydroxyterephthalate upon hydroxylation with hydroxyl radicals (Page et al., 2010). Production of hydroxyl radicals was monitored by measuring absorption at 315 nm with a Shimadzu UV-1800 spectrophotometer (Shimadzu, Kyoto, Japan). For every sample, the area under the absorbance vs. time curve was calculated as explained by Guckert et al. (1996). A non-degrading fungal strain and abiotic control were included as negative controls. Every condition was tested in triplicate.

### Biodegradation of Chlorendic Acid in Soil

Fungal degradation of chlorendic acid in soil was examined for the selected fungal isolates. Three different inoculation methods in soil were tested. For inoculation method A, the fungal isolates were cultivated on solid malt extract-peptone medium in Petri dishes of 15 mm at 23°C until the mycelia covered the entire surface of the Petri dishes. The solid medium covered with the mycelia was subsequently cut into small sections of 5 mm^2^ and the contents of three Petri dishes was mixed with 1.5 kg of unpolluted soil from the experimental field spiked with 50 mg chlorendic acid per kg soil, which was distributed over three pots of 500 g each. For the control condition, the contents of three Petri dishes with solid medium, without mycelia were used. For inoculation method B, the fungal isolates were cultivated in 100 ml liquid malt extract-peptone medium for 10 days at 23°C at 120 rpm, after which the mycelia were filtered out of the medium and 90 g was mixed under 1.5 kg of unpolluted soil from the field site spiked with 50 mg kg^–1^ chlorendic acid and distributed over three pots of 500 g each. Non-inoculated pots were used as a control. For inoculation method C, the fungal isolates were cultivated in 100 ml liquid malt extract-peptone medium for 10 days at 23°C at 200 rpm, leading to a more viscous culture, that was mixed under 1.5 kg of unpolluted soil from the field site spiked with 50 mg kg^–1^ chlorendic acid and distributed over three pots of 500 g each. For the control condition, 100 ml of non-inoculated medium was used. The pots were incubated at 23°C for 4 weeks and watered (25 ml) every other day, after which the concentrations of chlorendic acid in soils were determined. In an initial screening, only one soil sample per condition was analyzed, after which the remaining two replicates were analyzed for the conditions that showed the most promising results in the screening.

### Phytotoxicity Assay

To assess the toxicity of the intermediates formed during fungal degradation of chlorendic acid, a phytotoxicity test with *Arabidopsis thaliana* was performed using vertical agar plates. The selected fungi were grown in liquid malt extract-peptone medium for 1 week at 23°C and 120 rpm, subsequently washed twice in minimal Ingestad medium and transferred to minimal Ingestad medium enriched with 50 mg l^–1^ chlorendic acid and 3 mM glucose. After 2 weeks incubation at 23°C, the medium, containing the degradation products, was filter sterilized and 400 μl of this suspension was plated onto solid 1/4 Murashige and Skoog basal salt mixture medium in a 12 cm × 12 cm transparent plate (Murashige and Skoog, 1962). As a control, the selected fungi were grown in absence of chlorendic acid, after which the medium was filter sterilized and 400 μl was plated. This way, a distinction could be made between possible phytotoxic products formed by the fungi itself, unrelated to chlorendic acid degradation, and products formed due to fungal degradation of chlorendic acid.

*Arabidopsis thaliana* seeds were surface sterilized in 0.1% NaClO containing 0.1% Tween 80 for 1 min and subsequently washed 4 times in sterile distilled water. Seeds were dried under a laminar air flow and sown on solid 1/4 Murashige and Skoog medium plates supplemented with 15 mM sucrose. The plates were stored during 48 h at 4°C to ensure homogenous germination, after which they were placed vertically in a growth chamber with day/night temperatures of 22/18°C with a 12 h light and 12 h dark cycle, a relative humidity of 65% and a photosynthetic active radiation (PAR) that simulates the PAR in sunlight of 170 μmol m^–2^ s^–1^, provided by Philips GreenPower LED light modules. After 6 days, plants were transferred to the plates described above containing the fungal degradation products of chlorendic acid or the control media. After 2 weeks of growth, primary root length was determined using RootNav v1.8.1 (Pound et al., 2013). For every condition, 3 plates with each 5 plants were tested.

### Statistical Analyses

Statistical analyses were performed in R v3.0.0 (R Core Team, 2013). The Shapiro-Wilk test was used to evaluate normal distribution of the data and transformations were applied when needed to approximate normality. Homoscedasticity of variances was confirmed with a residuals plot. Significant differences were analyzed with an analysis of variances (ANOVA) followed by *post hoc* comparisons using the Tukey’s Honest Significant Differences multiple comparisons test.

### Sanger Sequence Submission

The obtained ITS-2 rRNA gene sequences of the fungi have been submitted to the NCBI database with the accession numbers MF631853-MF631857. The partial bacterial 16S rRNA gene sequences can be found in Genbank under the accession numbers MN103889-MN103942.

## Results and Discussion

### Chlorendic Acid-Degrading Bacteria and Fungi

**FIGURE 2:**
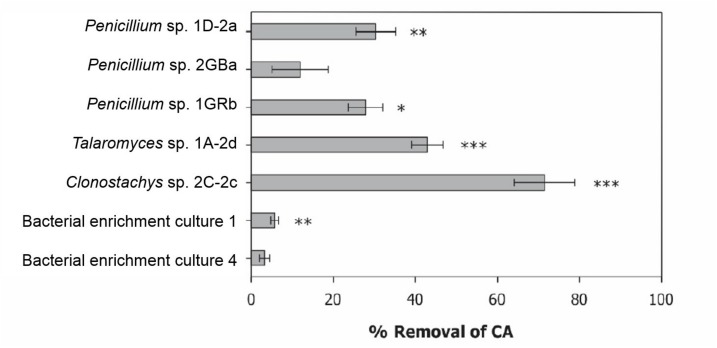
Degradation potential of selected bacterial consortia and fungal isolates. Displayed as percentage removal of chlorendic acid from the liquid phase relative to the abiotic control condition. CA, chlorendic acid. ^*^p < 0.05, ^∗∗^*p* < 0.01, and ^∗∗∗^*p* < 0.001 compared to the abiotic control using two-way ANOVA with Tukey’s HSD pairwise comparisons, *n* = 3.

At the end of the selective enrichments experiment, chlorendic acid removal reached 5% for two bacterial consortia, one and four (Figure 2). Consortium one consisted of three bacterial isolates, *Lysinibacillus* sp., *Achromobacter* sp. and *Paenibacillus* sp., and culture four consisted of two bacterial strains, *Lysinibacillus* sp. and *Brevundimonas* sp. In addition, 60 more strains were purified from soil, rhizosphere and root endophytic tissues, and 16S RNA gene Sanger sequenced (Supplementary Figure S1 and Supplementary Table S1). The degradation potential of these isolates was low, so further chlorendic acid degradation potential was focused on the fungi.

**FIGURE 3:**
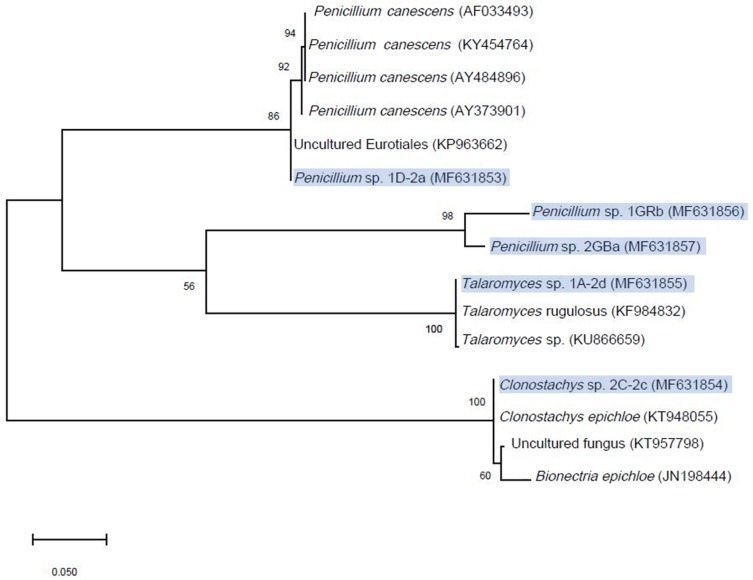
Maximum Likelihood phylogenetic tree of the isolated fungi compared to their closest relatives in the MycoBank. Scale-bar shows the number of substitutions per site, node labels show the percentage consensus support (%) and the tree was constructed with bootstrap resampling method and 500 replicates in MEGA X. Isolates described in this study are highlighted in blue.

In total 75 fungi were isolated from the contaminated soil of which sixteen isolates removed between 10 and 20% of chlorendic acid from the liquid phase and four removed between 20 to 50% of chlorendic acid (Figure 2). Of these four, *Clonostachys* sp. 2C-2c showed the highest capacity to degrade chlorendic acid, with 71% removal of chlorendic acid from the liquid phase. *Talaromyces* sp. 1A-2d showed the second highest capacity to degrade chlorendic acid with a 45% removal rate, followed by the isolates *Penicillium* sp. 1D-2a and *Penicillium* sp. 1GRb which showed 26% and 30% removal of chlorendic acid, respectively, compared to the abiotic control. The closest relatives of the degradative fungal isolates blasted against MycoBank are shown in the phylogenetic tree (Figure 3).

Bacteria are not the best in degrading chlorendic acid compared to the fungi. Bacteria generally use pollutants as co-substrates for growth during biodegradation compared to fungi (Harms et al., 2011). Since chlorendic acid is a highly chlorinated compound which gives it a low energetic value, can be the reason for the slow and insignificant degradation. Another possibility is that the bacteria lack the appropriate degradative machinery for this class of compounds. In fact, bacteria use specific biochemical pathways for degrading xenobiotic compounds that are most often modifications of existing ones (Harms et al., 2011). Those new pathways however, will only develop when there is a selective benefit for the bacteria in terms of sole carbon and energy source (Harms et al., 2011). Fungi can have an advantage in this situation, since they generally degrade pollutants by non-selective enzymes and radicals. Their enzymes both extracellular, like laccases and peroxidases, and cell-bound enzymes like cytochrome P450, are characterized by a low substrate specificity allowing them to co-metabolize a wide range of structurally different pollutants (Harms et al., 2011). Several studies already described the capacity of fungi to degrade compounds structurally related to chlorendic acid, such as pesticides aldrin and dieldrin (Fragoeiro and Magan, 2005; Kamei et al., 2010; Purnomo et al., 2017). It is worth mentioning that the three best degraders were isolated from soil at a depth of 2 m, where the concentration of chlorendic acid was the highest (39.33 mg kg^–1^). *Penicillium* spp. have already been reviewed for their capacity to degrade organic pollutants (Leitão, 2009; Pinedo-Rivilla et al., 2009; Maqbool et al., 2016) and were identified as degraders of hydrocarbons (Chaillan et al., 2004), terbuthylazine (Pinto et al., 2012), bensulphuron-methyl (Peng et al., 2012), chlorfenvinphos (Oliveira et al., 2015), and endosulfan, all structurally related to chlorendic acid (Mohanasrinivasan et al., 2013; Romero-Aguilar et al., 2014). Also, *Talaromyces* spp. have been investigated for their biodegradative capacities of hydrocarbons (Chaillan et al., 2004), biphenyl (Romero et al., 2005), nicosulfuron (Song et al., 2013), polycyclic aromatic hydrocarbons (PAHs) (Reyes-César et al., 2014), and textile dyes (Chatterjee et al., 2017). *Clonostachys* spp. have been less well known for biodegradation of xenobiotic compounds, however, Cox et al. (1993) isolated a styrene utilizing *Clonostachys rosea* strain for use in biofiltration of hydrocarbon polluted air. Because of their higher degradation potential than the bacteria, the five fungal isolates were selected for further degradation and biosorption experiments, while the bacteria were further tested for plant growth-promotion potential.

### Biodegradation vs. Biosorption

**FIGURE 4:**
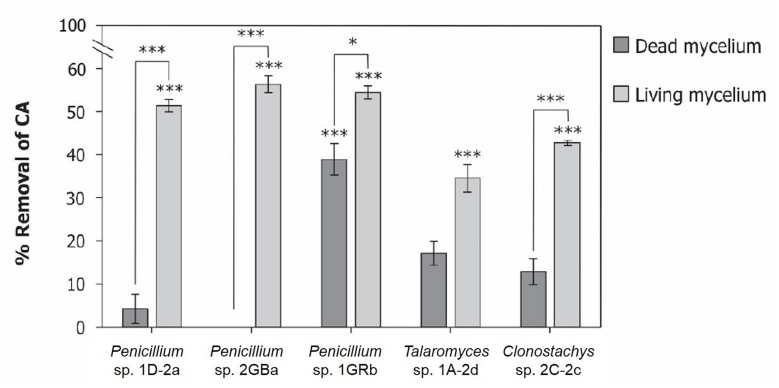
Biosorption vs. biodegradation of chlorendic acid by selected fungal isolates. Displayed as percentage removal of chlorendic acid from the liquid phase relative to the abiotic control condition. CA, chlorendic acid. Significance stars above bars represent significant differences compared to abiotic control. Significance stars between bars represent significant differences between dead and alive mycelia per fungal strain. ^*^p < 0.05, ^∗∗∗^*p* < 0.001 using two-way ANOVA with Tukey’s HSD pairwise comparisons, *n* = 3.

The four selected fungal isolates showed the capacity to remove chlorendic acid from the liquid phase. This could either be the result of biodegradation or of biosorption. Biosorption is mediated by a metabolism-independent processes in the cell wall of the fungal mycelia, while biodegradation is mediated by oxidation via anaerobic or aerobic metabolism (Aksu, 2005). To identify the main mechanism of chlorendic acid removal from the liquid phase by the selected fungi, the removal capacity of living fungi was compared with that of their dead counterparts (Figure 4). All living fungal isolates significantly removed chlorendic acid from the liquid phase, while with the dead mycelia this was only the case for 1GRb. Moreover, a considerably higher chlorendic acid removal was observed for living mycelia compared to their dead counterparts, except for *Talaromyces* 1A-2d. These findings suggest that a significant percentage of the removal of chlorendic acid from the liquid phase could be attributed to biodegradation of chlorendic acid by the fungi. For *Penicillium* 1D-2a and *Penicillium* 2GBa the fraction attributed to biodegradation was highest.

To our knowledge, this is the first report of direct biological degradation of chlorendic acid, with the first isolation and identification of chlorendic acid-degrading microorganisms. This is in contradiction to earlier studies, that describe chlorendic acid as resistant to biological degradation (Sebastian et al., 1996; Ndokiari, 2013).

### Toxicity of Degradation Intermediates

**FIGURE 5:**
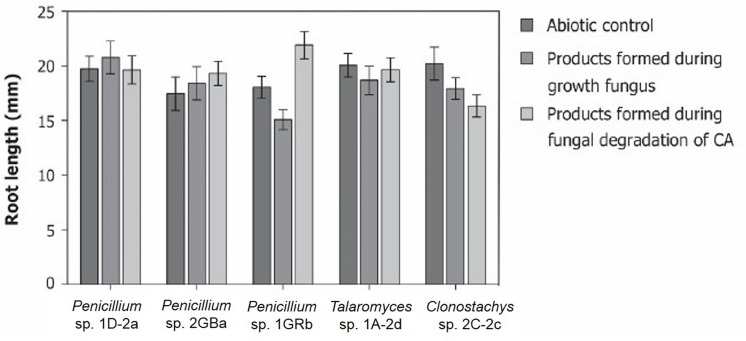
Phytotoxicity of possible degradation products formed during fungal degradation of chlorendic acid. Presented as effect of products on root length of *A. thaliana*. CA, chlorendic acid. ^∗∗∗^*p* < 0.001 using two-way ANOVA with Tukey’s HSD pairwise comparisons, *n* = 15.

The degradation pathways of chlorendic acid that are followed during biodegradation by the selected fungal strains are unknown. Because of the difficulties relating to both extracting and measuring unknown intermediate degradation products, another experiment was conducted to obtain an idea of the possible toxicity of the intermediate degradation products. Therefore, the model plant *A. thaliana* was used on which the phytotoxicity of the products formed during fungal degradation of chlorendic acid was tested and compared to that of the products formed during normal fungal growth (in absence of chlorendic acid) and to the abiotic control condition (Figure 5). For all fungi tested, the products formed during growth on CA had no negative effects on plant root growth (Figure 5). Therefore, we may conclude that the formed degradation products are not harmful to plants.

### Degradation Mechanism of Chlorendic Acid

The activity of extracellular enzymes (laccases and peroxidases) was determined for the four fungal isolates. However, no activity of either laccases or peroxidases was detected. However, fungi can also generate Fenton-mediated hydroxyl radicals, which are powerful non-selective oxidants, via secretion of iron reducing compounds. Upon reduction of Fe^3+^ to Fe^2+^ Fenton chemistry is induced, leading to production of hydroxyl radicals (Hammel et al., 2002). Induction of Fenton chemistry by fungi was originally studied in brown-rot fungi (Koenigs, 1974; Hyde and Wood, 1997; Arantes et al., 2011, 2012), but is also described for white rot fungi (Arantes et al., 2011) and ectomycorrhizal fungi (Rineau et al., 2012; Shah et al., 2015), where the generated hydroxyl radicals oxidize lignified plant cell walls or decompose organic matter. Hydroxyl radicals can degrade organic pollutants, because of their strong oxidative capacity (Freshour et al., 1996). Production of hydroxyl radicals is usually achieved via ozonation or UV-radiation. Several studies proved degradation of chlorendic acid by formation of radicals via chemical oxidation, like ozonation (Freshour et al., 1996; Sebastian et al., 1996; Hermes and Knupp, 2015), UV-radiation (Ndokiari, 2013), or gamma irradiation (Shah et al., 2016).

**FIGURE 6:**
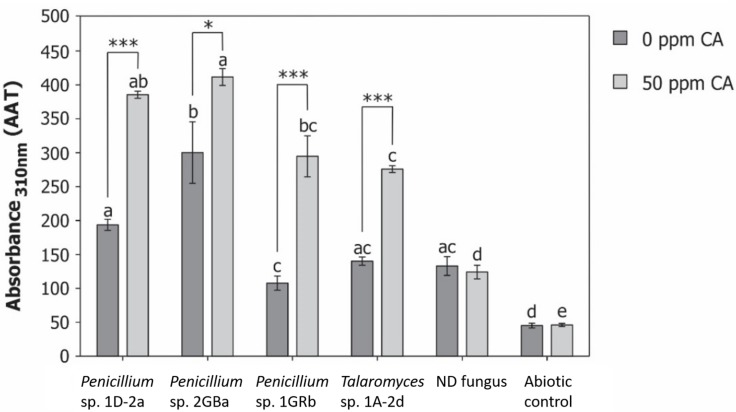
Production of hydroxyl radicals during degradation of chlorendic acid. Displayed as area under absorbance vs. time curve (AAT). ND, non-degrading fungus; CA, chlorendic acid. Letters above bars indicate significant differences (*p* < 0.05) within the same condition (with or without chlorendic acid). Significance stars indicate significant differences between fungal isolates in presence and absence of chlorendic acid. ^*^p < 0.05, ^∗∗∗^*p* < 0.001 using two-way ANOVA with Tukey’s HSD pairwise comparisons, *n* = 15.

In a preliminary experiment, we observed 50% degradation of chlorendic acid when exposed to UV-radiation. These findings strongly indicate that free radicals are involved in the degradation of chlorendic acid by fungal isolates. Therefore, the selected fungi were tested for their production of hydroxyl radicals during degradation of chlorendic acid (Figure 6). In absence of chlorendic acid, all fungi produced significantly more hydroxyl radicals compared to the abioticabio control. Compared to the non-degrading control, only *Penicillium* 2GBa showed a significant increase of hydroxyl radicals production. In presence of chlorendic acid, all fungi demonstrated significant increases in hydroxyl radical production compared to the abiotic control and all degrading fungi produced significantly more hydroxyl radicals than the non-degrading fungus. Moreover, the degrading fungi significantly increased hydroxyl radicals production in presence of chlorendic acid. This indicates that during degradation of chlorendic acid by the fungal isolates, the production of hydroxyl radicals was increased, which could be the mechanism behind the degradation. Earlier studies already reported degradation of xenobiotic compounds by a fungal induced Fenton chemistry in *Gloeophyllum* spp. (polyethylene glycol, Kerem et al., 1999; dichlorophenol, Schlosser et al., 2000; 2,4,6-trinitrotoluene, Newcombe et al., 2002; 2-fluorophenol, Kramer et al., 2004; DDT, Purnomo et al., 2008; polystyrene sulfonate, Krueger et al., 2015). Formic acid, acetic acid, chloride and chlorate were detected as degradation products during chemical ozonation of chlorendic acid (Hermes and Knupp, 2015). We did not detect any of these degradation compounds in the culture supernatans, though follow-up degradation studies with mutants are needed to identify which intermediates are formed.

### Biodegradation of Chlorendic Acid in Soil

Four of the five selected chlorendic acid-degrading fungi were examined for their capacities to degrade chlorendic acid in soil. *Clonostachys* was left out because it grew very slow. Three different inoculation methods were tested in a first screening (Table 1). Applying inoculation methods B and C, the highest percentages of degradation were obtained, with 33% of degradation by *Penicillium* 1D-2a and *Penicillium* 1GRb when using inoculation method B and 29% of degradation with *Penicillium* 2GBa when using inoculation method C. These were therefore further investigated and inoculation method B showed the best method, with *Penicillium* 1D-2a as the best chlorendic acid degrader in soil (42% degradation ± 8), followed by *Penicillium* 1GRb and incoculation method B (31% degradation ± 16), and third best *Penicillium* 2GBa with inoculation method C (15% degradation ± 8). Earlier studies with *Penicillium* spp. already described this genus as being able to degrade xenobiotic compounds in soil. As such, He et al. (2007) reported a *Penicillium* sp. that was degrading the herbicide metsulfuron-methyl in rhizospheric soil of wheat. Also, Peng et al. (2012) revealed biodegradation of bensulfuron-methyl by a *Penicillium pinophilum* strain in soil. Sondhia et al. (2013) demonstrated biodegradation of the herbicide pyrazosulfuron-ethyl in soil by *Penicillium chrysogenum*. The fact that chlorendic acid can be biologically degraded in soil opens perspectives for *in situ* applications with the selected fungal isolates.

**TABLE 1:** Degradation capacity of chlorendic acid in soil by different fungal isolates.

	**% degradation of CA**		
	
	**Inoculation method A**	**Inoculation method B**	**Inoculation method C**
*Penicillium* sp. 1D-2a	15 ± 3	**33 ± 5**	0
*Penicillium* sp. 2GBa	0	4 ± 0.5	**29 ± 5**
*Penicillium* sp. 1GRb	0	**33 ± 7**	0
*Talaromyces* sp. 1A-2d	19 ± 2	4 ± 1	0

Displayed as percentage degradation of chlorendic acid relative to the non-inoculated control condition, using three different inoculation methods^*^, *n* = 3. ^*^Inoculation method A, involved mixing 5 mm^2^ pieces of mycelia from three Petri dishes with 1.5 kg of CA-spiked soil. For inoculation method B, 90 g of fungi grown in liquid malt extract-peptone medium for 10 days at 23°C and 120 rpm, was mixed under 1.5 kg of CA-spiked soil. Inoculation method C was similar to B, except that cultures were shaken at 200 rpm instead of 120 rpm leading to a more viscous culture, that was mixed under 1.5 kg of CA-spiked soil. Pots were incubated at 23°C for 4 weeks and watered (25 ml) every other day, after which the concentrations of CA in soils were determined. Bold values are the most promising results. These conditions were further tested.

### Plant Growth-Promoting Capacity of the Bacteria

The cultivable bacteria that were isolated from the polluted site were screened for plant growth-promoting traits *in vitro* including phosphorous solubilization, nitrogen fixation, production of indole-3-acetic acid (IAA), siderophores, acetoin, organic acids, and 1-aminocyclopropane-1-carboxylic acid (ACC) deaminase activity (Supplementary Figure S1 and Supplementary Table S1). Sixty-one bacterial strains were selected and tested and showed positive response for at least one of the plant growth promotion tests (Supplementary Table S1). Although bacteria were not the most potent degraders, they can via indirect mechanisms contribute to enhanced phytoremediation by stimulating tree growth, biomass, root development, stress reduction and nutrient acquisition. Future inoculation studies can show their full potential *in planta* when inoculated on the field site.

## Conclusion

Both bacteria and fungi were isolated and identified from the polluted site and our results indicate that the isolated fungi showed much higher capacities for chlorendic acid degradation than the isolated bacteria. The suggested mechanism of degradation is through generation of hydroxyl radicals by inducing Fenton chemistry. Three of the five fungal isolates were identified as *Penicillium* spp., one as *Talaromyces* sp. and one as *Clonostachys*. These five fungal isolates are very promising for application in bioremediation of chlorendic acid. Moreover, since the degradative capacity of the generated hydroxyl radicals is not substrate specific, they could also be exploited for bioremediation of other organic pollutants. Most of the isolated bacteria showed plant growth-promoting capacities *in vitro* can be co-inoculatd with fungi in later stages for enhancing phytoremediation of chlorendic acid. This paper demonstrates that recalcitrant, seemingly non-biodegradable organic pollutants can be remediated making use of biological techniques.

## Author Contributions

IJ, ST, NW, and JV conceived and designed the study. JV and NW coordinated the execution of the project with IJ. IJ wrote the manuscript in collaboration with ST, FR, JV, and NW. JV was the promoter of the study. GT-F helped with the design and execution of the fungal enzyme assays. FR helped with the interpretation of the hydroxyl radical production assays. PS and RC provided technical and analytical support for the chlorendic acid HPLC analyses. All authors contributed to the elaboration of the study design, took part in reviewing the methods, each member contributed specifically to the parts of the study corresponding with their own expertise, and read and approved the final version of the manuscript.

## Conflict of Interest Statement

The authors declare that the research was conducted in the absence of any commercial or financial relationships that could be construed as a potential conflict of interest.
