# Supplementary material for: Fenton-Mediated Biodegradation of Chlorendic Acid – A Highly Chlorinated Organic Pollutant – By Fungi Isolated From a Polluted Site
Source: Front Microbiol. 2019 Aug 14;10:1892. doi: 10.3389/fmicb.2019.01892 (PMC6702520; doi:10.3389/fmicb.2019.01892)
Supplement: Supplementary file 1 [file Data_Sheet_1.docx]

**Supplementary Tables and Figures**

**Table S1: Plant growth-promotion traits of selected bacteria.** + means the strain scored positive on the corresponding test, – means it scored negative. P = phosphorous solubilization, N = nitrogen fixation, IAA = indole-3-acetic acid production, Sid = siderophores production, Acet = acetoin production, OA = organic acids production and ACC = aminocyclopropane-1-carboxylic acid deaminase activity.

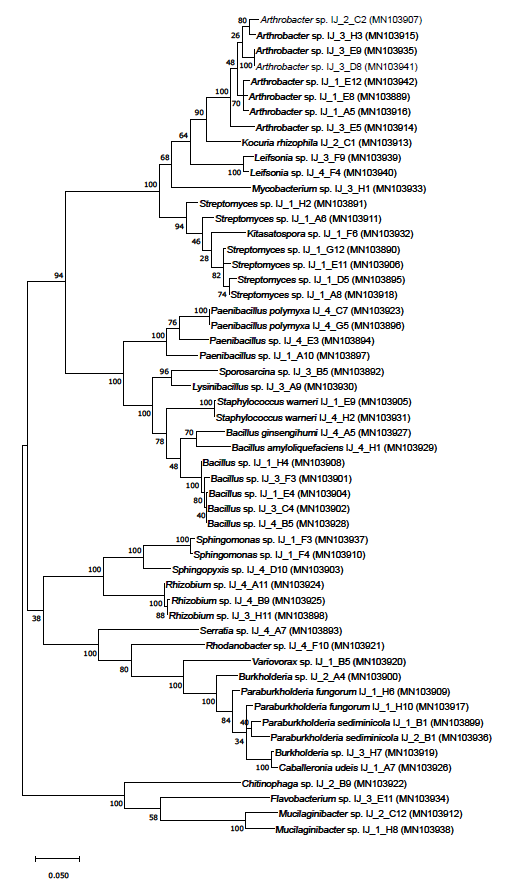


**Figure S1: Phylogenetic tree of the 16S rRNA gene sequences of the isolated bacteria using the Maximum Likelihood method and Tamura-Nei model** (Tamura K. and Nei M., 1993). The percentage of trees in which the associated taxa clustered together is shown next to the branches, based on 500 bootstrap replicates. Initial trees for the heuristic search were obtained automatically by applying Neighbor-Join and BioNJ algorithms to a matrix of pairwise distances estimated using the Maximum Composite Likelihood (MCL) approach in MEGA X, and then selecting the topology with superior log likelihood value. The tree is drawn to scale, with branch lengths measured in the number of substitutions per site. Bar shows 0.050 substitutions per nucleotide position. The analysis involved 54 nucleotide sequences. Codon positions included were 1st+2nd+3rd+noncoding. There were a total of 1373 positions in the final dataset. Evolutionary analyses were conducted in MEGA X (Kumar et al., 2018).
